# Comparative Phytochemical Analyses and Metabolic Profiling of Different Phenotypes of Chinese Cabbage (*Brassica Rapa* ssp. *Pekinensis*)

**DOI:** 10.3390/foods8110587

**Published:** 2019-11-19

**Authors:** Chang Ha Park, Hyeon Ji Yeo, Soo-Yun Park, Jae Kwang Kim, Sang Un Park

**Affiliations:** 1 Max-Planck-Institute of Molecular Plant Physiology, Am Müehlenberg 1, 14476 Potsdam-Golm, Germany; parkch804@gmail.com; 2 Department of Crop Science, Chungnam National University, 99 Daehak-Ro, Yuseong-gu, Daejeon 34134, Korea; guswl7627@gmail.com; 3 National Institute of Agricultural Sciences, Rural Development Administration, Wanju-gun, Jeonbuk 55365, Korea; psy22@korea.kr; 4 Division of Life Sciencesand Bio-Resource and Environmental Center, Incheon National University, Incheon 406-772, Korea

**Keywords:** *Brassica rapa* ssp. *pekinensis*, Chinese cabbage, primary metabolite, secondary metabolite, glucosinolate, phenolics, carotenoid

## Abstract

This study aimed to comprehensively examine the interface between primary and secondary metabolites in oval- and rectangular-shaped Chinese cabbage (*Brassica rapa* ssp. *pekinensis*) using gas chromatography coupled with time-of-flight mass spectrometry (GC-TOFMS) and high-performance liquid chromatography (HPLC). In addition to differences in shape, there was significant morphological variation between the two cultivars. The rectangular variety had greater height and deeper green color, whereas the oval variety had more leaves and greater width. A total of 42 primary metabolites identified by GC-TOFMS were subjected to partial least-squares discriminant, which indicated significant differences in the primary and secondary metabolisms of the two cultivars. Furthermore, total glucosinolate and phenolic contents were higher in the oval cultivar, whereas the rectangular cultivar contained a higher level of total carotenoids. This metabolome study comprehensively describes the relationship between primary and secondary metabolites in the oval and rectangular cultivars of Chinese cabbage and provides information useful for developing strategies to enhance the biosynthesis of glucosinolates, phenolics, and carotenoids in Chinese cabbage. Additionally, this work highlights that HPLC and GC-TOFMS–based metabolite profiling is suitable techniques to determine metabolic differences in Chinese cabbage.

## 1. Introduction

More than 200 naturally occurring glucosinolates have been identified in *Brassicaceae* species [1]. These glucosinolates have been assigned to three classes—aliphatic, aromatic, and indolic glucosinolates—derived from the amino acids methionine, phenylalanine, and tryptophan, respectively. Myrosinase is an endogenous enzyme that hydrolyzes glucosinolates to yield various bioactive chemicals. The resulting products, such as epithionitriles, nitriles, substituted isothiocyanates, oxazolidinethiones, and thiocyanates, are associated with plant defense and possess a broad range of biological properties related to human health, such as protective effects against cancers of the breast, colon, rectum, lungs, stomach, prostate, and pancreas [2,3,4].

Phenylpropanoids, found mainly in most higher plants, are plant secondary metabolites possessing potent biological activities that play a role in the protection of plants against biological, physical, and environmental stresses, including insect and pathogen attack, wounding, and exposure to excess light, excess ultraviolet radiation, and low and high temperature [5,6]. Furthermore, the consumption of plant foods containing various phenylpropanoids are possibly beneficial to human health, since previous studies have indicated that dietary phenolics possess cancer-prevention, cardiovascular disease prevention, anti-estrogenic, antioxidant, and antimicrobial activities [2,3].

Carotenoids, derived from a terpenoid precursor, are an important group of secondary metabolites representing a broad range of more than 600 naturally occurring yellow, orange, and red pigments that accumulate in plant plastids [7] and play an important role in numerous physiological processes in plants. For instance, carotenoid pigments act as light harvesters in photosynthetic membranes and inhibit photooxidation. Additionally, the flower and fruit colors derived from carotenoid pigments attract seed dispersal agents and pollinators [8]. Furthermore, carotenoid pigments are the precursors of abscisic acid, a plant hormone related to the regulation of stress and developmental processes [9]. In humans, several carotenoids are essential nutrients, since humans are unable to synthesize carotenoids from endogenous precursors and can only obtain them from dietary sources. Others carotenoids contribute to the prevention of several diseases; i.e., dietary carotenoids are associated with a reduced risk of cancer, immune disorders, degenerative diseases, and age-related diseases [2,3].

**Figure 1:**
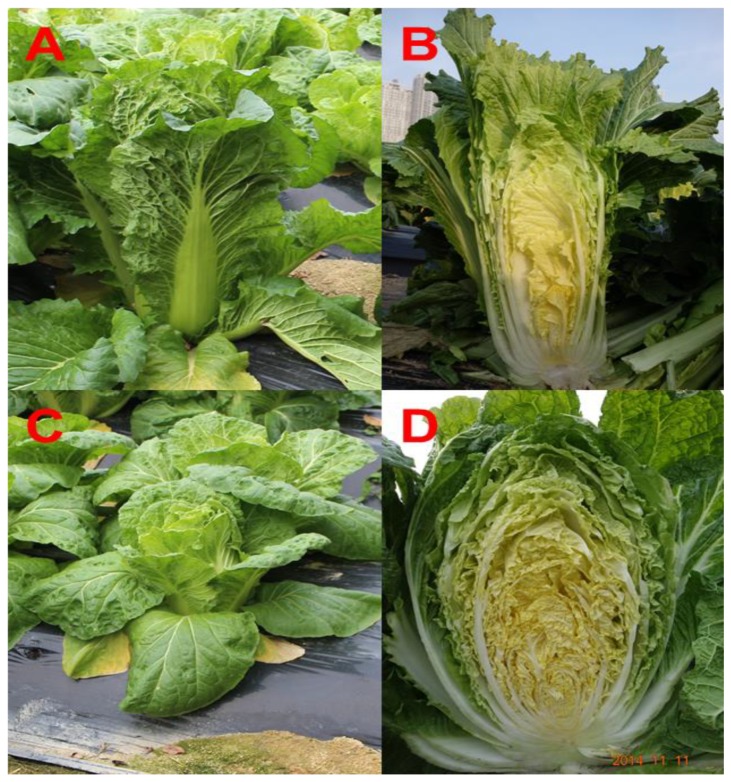
Two different phenotypes of Chinese cabbage. (**A**) Rectangular Chinese cabbage; (**B**) longitudinal section of the rectangular Chinese cabbage; (**C**) oval Chinese cabbage; (**D**) longitudinal section of the oval Chinese cabbage.

Chinese cabbage (*Brassica rapa* L. subsp. *pekinensis*) is a leafy green vegetable that is grown biennially and includes various cultivars exhibiting different phenotypes, such as those associated with color and shape. In this study, oval and rectangular types of Chinese cabbage were used as plant samples (Figure 1). We focused on profiling and quantifying the variety of metabolites in these cultivars of Chinese cabbage, using gas chromatography coupled with time-of-flight mass spectrometry (GC-TOFMS) and high-performance liquid chromatography (HPLC), and comprehensively determine connections between the identified metabolites. To our knowledge, no previous study has examined differences between the primary and secondary metabolites in oval and rectangular cultivars of Chinese cabbage.

## 2. Materials and Methods

### 2.1. Plant Materials

Seeds of Chinese cabbage (*B*. *rapa* subsp. *pekinensis* cv. Miss Jin) exhibiting rectangular shape and Chinese cabbage (*B*. *rapa* subsp. *pekinensis* cv. Asia Spring) exhibiting oval shape were purchased from Asia Seed Co., Ltd. (Seoul, Korea). The seeds of both Chinese cabbage cultivars were cultivated under field conditions at an experimental farm in Icheon, Gyeonggi-do, Korea. Three independent plants were prepared for each cultivar of Chinese cabbage. The Chinese cabbage (Figure 1) plants were harvested in triplicate after 85 day and immediately frozen in liquid nitrogen at −196 °C. All the samples were then stored in deep freezer at −80 °C and lyophilized for HPLC and GC-TOFMS analyses. All chemical analyses were carried out in triplicate.

### 2.2. GC-TOFMS Analysis

Hydrophilic metabolite extraction was carried out using a previously described method [10]. Powdered Chinese cabbage (0.01 g) was extracted with 1 mL of a water/chloroform/methanol (1:1:2.5 *v/v/v*) mixture, followed by addition of 60 µL of ribitol (0.2 g L^−1^) as an internal standard. The extraction was carried out at 37 °C for 30 min in a compact thermomixer at a mixing frequency of 1200× *g*. After centrifugation at 10,000× *g* for 5 min, 0.8 mL of the polar phase was transferred to a fresh tube, to which 0.4 mL of distilled water was added. The mixture was then centrifuged at 10,000× *g* for 5 min. The methanol/water phase was evaporated in a CVE-2000 centrifugal concentrator (Eyela, Japan) for 2 h, and the remaining material was then lyophilized using an FD8512 freeze-dryer (Ilshin Lab Co., Ltd., Daejeon, Korea) for 16 h. The lyophilized residues were subsequently processed in two stages—methoxime derivatization and trimethylsilyl etherification. Methoxyamine hydrochloride/pyridine (80 μL, 20 g L^−1^) was added to the vial, which was shaken at 30 °C for 90 min. After centrifuging to settle the solution to the bottom of the vial, 80 μL of *N*-methyl-*N*-(trimethylsilyl)trifluoroacetamide was added, followed by heating at 37 °C for 30 min. GC-TOFMS was performed using an Agilent 7890A gas chromatograph (Agilent, Atlanta, GA, USA) combined with a Pegasus HT TOF mass spectrometer (LECO, St. Joseph, MI, USA). The gas chromatograph was equipped with a CP–Sil 8 CB low bleed/MS fused-silica capillary column (5% phenyl/95% dimethylpolysiloxane, 60 m × 0.25 mm ID, 0.25 μm film thickness; Varian Inc., Palo Alto, CA, USA). The operating conditions were set as follow: injection port temperature, 230 °C; helium gas flow rate, 1.0 mL min^−1^; split ratio, 1:25. The temperature program was set as follows: initial temperature of 80 °C, 2 min; an increase to 320 °C at 15 °C/min; 10 min heating at 320 °C; transfer line temperature, 250 °C; the ion source temperatures, 200 °C; the scanned mass range, m/z 85−600; and the detector voltage, 1700 V. The values represent the means ± SD of three biological replicates (Figure S1).

### 2.3. Glucosinolate Extraction and HPLC Analysis

Glucosinolates were extracted and analyzed using previously reported procedures with slight modification [1]. Freeze-dried cabbage powder (0.1 g) was extracted with 1.5 mL of 70% (*v*/*v*) boiling aqueous methanol at 70 °C for 5 min in a water bath (HB-205 WP; KOREA QUALITY MACHINE SCIENCE, Seoul, Korea) and then centrifuged at 14,000× *g* at 4 °C for 10 min. The supernatant was collected into a 15-mL conical tube (H20015; Hyundai Micro Co., Ltd. Seoul, Korea), and the resultant pellets were re-extracted a further two times using the same procedure. The collected supernatants, as the crude glucosinolate extracts, were carefully loaded onto a mini-column containing DEAE-Sephadex A-25 (GE Healthcare, Uppsala, Sweden) and rinsed with 3 ml of distilled water. The elute was then desulfated by addition of 75 μL of purified arylsulfatase and incubated at room temperature overnight. Thereafter, desulfated glucosinolate samples were eluted with 0.5 mL (×3) of ultrapure water into a 2.0-mL safe-lock microcentrifuge tube (Eppendorf, Seoul, Korea) and passed through 0.22-μm PTFE syringe filters (Sterlitech Corp., Kent, WA, USA) into a brown vial. The HPLC analysis condition, system, and gradient program were taken from previous studies [1,11]. The external standard, sinigrin monohydrate from horseradish (99%), was purchased from Sigma-Aldrich Co., Ltd. (St. Louis, MO, USA). Each glucosinolate was identified by comparison with the retention times of the HPLC chromatograms in our previous papers [1,12] and quantitated based on HPLC retention times, HPLC areas, and response factors compared with the external standard, sinigrin (0.1 g L^−1^) (Figure S1).

### 2.4. Phenolic Compound Extraction and HPLC Analysis

A previously reported method was used to extract and analyze phenylpropanoid compounds [13]. The external standards trans-cinnamic acid, catechin hydrate, benzoic acid, rutin, chlorogenic acid, caffeic acid, epicatechin. and ferulic acid were purchased from Sigma-Aldrich Co., Ltd. (St. Louis, MO, USA), whereas hydroxybenzoic acid was purchased from Extrasynthese (Genay, France). Hydrochloric acid and acetic acid were purchased from SAMCHUN Chemical (Pyeongtaek, Korea). Methanol was purchased from J.T Baker^®^ Chemicals (Phillipsburg, NJ, USA), respectively. As the first step in the extraction procedure, 6 mL of aqueous methanol (62.5%, *v*/*v*) containing 2 g/L *tert*-butylhydroquinone was added to accurately weighed 0.1 g of sample powders of both oval and rectangular types of Chinese cabbage. For hydrolysis, 1.5 mL of 6 N hydrochloric acid was added to each sample, followed by incubation in a water bath set at 90 °C for 2 h. The crude extracts were then centrifuged at 1000× *g* for 10 min, and the supernatant was then diluted two-fold. The extracts were then passed through 0.45-μm PTFE syringe filters (Millipore, Bedford, MA, USA) and transferred to 1.8-mL brown glass vials prior to HPLC-UV analysis. The extraction procedure was performed a further two times for a total of three extractions. For identification and quantitation of the phenylpropanoid compounds in each sample, the HPLC analysis condition, system, and gradient program were taken from Kim et al. [13]. Individual phenolics were identified based retention times and spiking experiments and quantitated with reference to a corresponding calibration curve (Figure S1).

### 2.5. Carotenoid Extraction and HPLC Analysis

Sample preparation and HPLC analysis of carotenoids were performed according previously described methods [14]. Powders (0.3 g) of both oval and rectangular types of Chinese cabbage were transferred to 15-mL conical tubes, followed by the addition of 3 mL of ascorbic acid/ethanol (0.1%, *w/v*). The mixture was strongly vortex for 20 s and then incubated in a water bath set at 85 °C for 5 min. Saponification was then performed by the addition of 120 μL of potassium hydroxide (80 %, *w/v*) to remove any potentially interfering oils. After an incubation at 85 °C for 10 min, each tube was placed on ice for 5 min to stop the reaction, and then an internal standard, 50 μL of β-apo-8′-carotenal (25 ppm) was added to the samples. Subsequently, 1.5 mL of cold distilled water and 1.5 mL of hexane (SAMCHUN Chemical, Pyeongtaek, Korea) were added to the sample, and the mixtures were centrifuged at 4 °C at 140× *g* to acquire supernatants from the layers. The extraction procedure was repeated twice for a total of three extractions. The collected aliquots were subsequently dried at room temperature under nitrogen gas in a fume hood, and the dried samples were then resuspended in 300 μL of dichloromethane/methanol (50:50 *v/v*). The final extracts were filtered through 0.45-μm PTFE syringe filters into brown-colored vials. The HPLC analysis condition, system, and gradient program were taken from Tuan et al. [14]. Individual carotenoids were identified with the help of our previous guidelines and through the combined use of the retention time and co-elution with β-apo-8′-carotenal, as an internal standard, and quantitated with reference to the corresponding calibration curves (Figure S1).

### 2.6. Chlorophyll Contents Analysis

Powders (0.1 g) of Chinese cabbage cultivars were transferred to 15-mL conical tubes, followed by the addition of 2 mL methanol. The mixture was strongly vortex for 20 s and then sonicated in a water bath for 30 min. After centrifugation and filtration, the absorbance of the extracts was measured at 665 and 652 nm to calculate the chlorophyll content using the formula mentioned by Lichtenthaler et al. [15].

### 2.7. Statistical Analysis

The data acquired from GC-TOFMS was scaled to unit variance scaling and then subjected to partial least squares-discriminant analysis (PLS-DA) using SIMCA-P software (version 12.0, Umetrics, Umeå, Sweden) to distinguish differences in metabolite profiles among the varieties (Table S1). PLS-DA output was presented as sore plot to exhibit the contrast. SPSS 24.0 (IBM, Chicago, IL, USA) was exploited to perform *t*-test to determine significance and depict metabolite map.

## 3. Results

### 3.1. Morphological Variation between Oval and Rectangular Chinese Cabbage

There was a significant variation between the oval and rectangular Chinese cabbage with regards to morphological characters (Table S2). The height of the rectangular cultivar (48.80 cm) was almost twice that of the oval cultivar (25.25 cm), and its leaves were of a deeper green color. The oval cultivar, however, showed higher values for other morphological variables. The width of the oval cultivar was 18.75 cm, which was 1.54 times wider than the rectangular cultivar. The number of leaves in the oval cultivar was 64, which was 24.4 leaves more than that of the rectangular cultivar. The head weight of the oval cultivar was 2.21 kg, whereas that of the rectangular cultivar was 2.12 kg.

### 3.2. Metabolite-Specific Profiling in Oval and Rectangular Chinese Cabbage

Identification of low-molecular weight molecules from the two Chinese cabbage cultivars was performed using GC-TOFMS. ChromaTOF was used to confirm peak location using an in-house library and comparing with reference compounds [10]. Furthermore, several metabolites were identified directly by comparing the sample mass chromatograms with the equivalents of external standard chemicals. A total of 42 metabolites (1 amine, 3 sugar alcohols, 7 sugars, 17 amino acids, and 14 organic acids) were detected in the oval Chinese cabbage. However, six of the amino acids detected in the oval cultivar (arginine, aspartic acid, leucine, isoleucine, proline, and threonine) were not detected in the rectangular Chinese cabbage. Quantitation was carried out using selected ions, as indicated in Table S3. Specifically, the concentrations of identified amino acids, such as alalnine, valine, serine, leucine, proline, glycine, threonine, beta-alanine, aspartic acid, pyroglutamic acid, 4-aminobutyric acid, arginine, glutamic acid, phenyalanine, asparagine, and glutamine, were significantly higher in the oval cultivar. Furthermore, this oval cultivar also contained larger amounts of tricarboxylic acid (TCA) cycle intermediates, including succinic acid, citric acid, fumaric acid, and malic acid. In contrast, the levels of fructose, raffinose, maltose, xylose, mannitol, inositol, and glycerol were higher in the rectangular cultivar (Figure S2).

### 3.3. Glucosinolates in the Oval and Rectangular Chinese Cabbage

HPLC analyses of the oval- and rectangular-shaped Chinese cabbage revealed eight glucosinolates (Table 1). The levels of these glucosinolate compounds varied between the two cultivars of Chinese cabbage. Total glucosinolate contents ranged from 2577.79 to 2896.78 μg g^−1^ dry weight in the Chinese cabbages and was 318.99 μg g^−1^ dry weight higher in the oval cultivar than in the rectangular cultivar. Among the individual glucosinolates, the level of progoitrin was approximately similar in the two cultivars. The levels of glucoalyssin, glucobrassicanapin, gluconapin, and 4-methoxyglucobrassicin were higher in the oval cultivar than in the rectangular cultivar. The amounts of gluconapin and glucobrassicanapin were 3.0 and 2.43 times higher, respectively, in the oval cultivar than in the rectangular cultivar. The levels of glucoalyssin and 4-methoxyglucobrassicin, were slightly higher in the oval cultivar. In contrast, the levels of 4-hydroxyglucobrassicin, glucobrassicin, and neoglucobrassicin were higher in the rectangular cultivar (2.18, 1.44, and 1.18 times higher, respectively). Interestingly, the contents of aliphatic glucosinolates, such as glucoalyssin, glucobrassicanapin, and gluconapin, were higher in the oval cultivar. However, with the exception of 4-methoxyglucobrassicin, the levels of indolic glucosinolates, including glucobrassicin, 4-hydroxyglucobrassicin, and neoglucobrassicin, were higher in the rectangular cultivar. From our study, we identified variations in the glucosinolate contents of the two different cultivars of Chinese cabbage.

### 3.4. Phenolics in the Oval and Rectangular Chinese Cabbage

Analyses of the rectangular and oval cultivars of Chinese cabbage confirmed the presence of the following nine phenolics: 4-hydroxybenzoic acid, catechin hydrate, chlorogenic acid, caffeic acid, epicatechin, ferulic acid, benzoic acid, rutin, and *trans*-cinnamic acid (Table 2). Among these, neither catechin hydrate nor rutin were detected in the rectangular cultivar of Chinese cabbage. The amounts of phenolics were observed to be higher in the oval cultivar, which contained a total amount of 226.53 μg g^−1^ dry weight phenolic compounds (1.17-fold higher than that in the rectangular Chinese cabbage). It was also observed that the oval cultivar contained the highest levels of *p*-hydroxybenzoic acid, catechin hydrate, chlorogenic acid, ferulate, rutin, and *trans*-cinnamate. Interestingly, catechin hydrate and rutin were only present in the oval cultivar. In both oval and rectangular cultivars of Chinese cabbage, the levels of *p*-hydroxybenzoic acid, chlorogenic acid, epicatechin, and ferulate were considerably higher than those of the other phenolic compounds. The level of ferulate in the oval cultivar was almost double that in the rectangular cultivar, whereas the levels of *p*-hydroxybenzoic acid, chlorogenic acid, and *trans*-cinnamate were 1.32, 1.08, and 1.68 times higher, respectively, in the oval cultivar. In contrast, benzoic acid was only present in the rectangular cultivar. The rectangular cultivar also had a 1.48 times higher level of caffeic acid than the oval cultivar.

### 3.5. Carotenoids and Chlorophylls in the Oval and Rectangular Chinese Cabbage

HPLC analyses of the oval and rectangular Chinese cabbage revealed the following nine types of carotenoids: violaxanthin, antheraxanthin, lutein, zeaxanthin, β-crytoxanthin, 13-*cis*-β-carotene, α-carotene, β-carotene, and 9-*cis*-β-carotene (Table 3). The amounts of total carotenoids were considerably higher in the rectangular cultivar of Chinese cabbage than in the oval cultivar. Specifically, the total amount of carotenoids contained in the rectangular cultivar (1643.13 μg g^−1^ dry weight) was 2.22-fold higher than that in the oval cultivar (741.73 μg g^−1^ dry weight). Among the individual carotenoids, the production of lutein and β-carotene was 2.40- and 2.24-fold higher in the rectangular cultivar, even though both cultivars accumulated large amounts of carotenoids. The rectangular cultivar also contained 1.64-, 1.76-, 1.40-, 3.06-, 2.43-, 1.39-, and 2.07-fold higher amounts of violaxanthin, antheraxanthin, zeaxanthin, β-crytoxanthin, 13-*cis*-β-carotene, α-carotene, and 9-*cis*-β-carotene, respectively, compared to the oval cultivar. Also, the amounts of total chlorophylls were significantly higher in rectangular cultivar. In particular, the level of chlorophyll b was 2.56-fold higher than that in the oval cultivar (Table 4).

### 3.6. A metabolite Map Comparing Primary and Secondary Metabolites in Oval and Rectangular Chinese Cabbage

**Figure 2:**
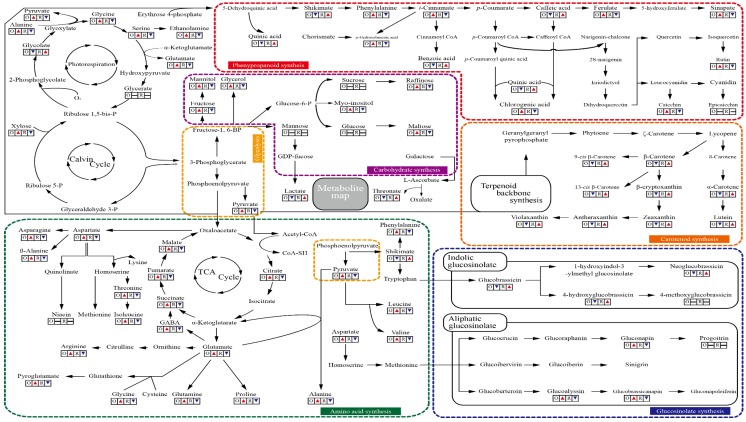
A metabolite map comparing primary and secondary metabolites of the oval and rectangular Chinese cabbage cultivars. The up arrow (▲) indicates that the mean value of the target metabolite is significantly higher (at *p* < 0.05). The down arrow (▼) indicates that the value of the metabolite is significantly lower (at *p* < 0.05). The horizontal bar (–) means that mean values of the target compounds were not significantly different (at *p* < 0.05). A different letter (O and R) indicated oval Chinese cabbage (B. rapa subsp. pekinensis cv. Asia Spring) and rectangular *Chinese cabbage* (Chinese cabbage. *B*. *rapa* subsp. *pekinensis* cv. Miss Jin).

A metabolite map was produced using the GC-TOFMS data of the 42 metabolites (Figure S2), and the HPLC data for eight glucosinolates (Table 1), nine phenolics (Table 2), and nine carotenoids (Table 3), to determine metabolic connection after *t*-test in Figure 2. Sugars are the most abundant metabolite in both cultivars. The comparison of carbohydrate levels between the oval and rectangular Chinese cabbage cultivars indicated that the total carbohydrate levels were similar in the both cultivars. Specifically, the levels of carbon sources, including fructose, mannitol, xylose, raffinose, and maltose, were statistically higher in the oval cultivar than in the rectangular cultivar. In contrast, the quantities of sucrose, mannose, and glucose were similar in the two cultivars (Figure S2). In the present study, a total of 17 amino acids were detected in the oval cultivar, whereas only 11 amino acids were identified in the rectangular cultivar, which lacked the amino acids leucine, isoleucine, proline, threonine, aspartate, and arginine present in the oval cultivar. The total amount of amino acids in the oval cultivar was significantly higher (3.39-fold) than that in the rectangular cultivar. The individual amounts of most amino acids were also higher in the oval cultivar compared with the rectangular cultivar; the exception being shikimate, which was 2.53-fold higher in the rectangular cultivar. The larger pools of glutamate (4.47-fold), glutamine (8.99-fold), and asparagine (13.64-fold), which play critical roles in nitrogen metabolism, in the oval cultivar led to increased amounts of other amino acids, compared with those in the rectangular cultivar. The greater abundance of alanine (5.34-fold), which is involved in photorespiration, reflected the higher levels of photorespiratory intermediates, including glycine (4.88-fold) and serine (1.70-fold), in the oval cultivar. However, the glycolate level (1.32-fold) was higher in the rectangular cultivar. Moreover, the higher amount of serine was related to a higher production of ethanolamine, which is involved in phospholipid metabolism. The level of pyruvate synthesized from glycolysis was 3.30-fold higher in the oval cultivar, and was associated with enhanced levels of alanine, leucine, and valine (9.48-fold) in comparison with the rectangular cultivar. A total of four intermediates of the TCA cycle were identified in both cultivars. The oval cultivar had larger pools of citrate (2.32-fold), succinate (4.04-fold), fumarate (1.64-fold), and malate (1.16-fold), which were associated with the level of pyruvate and higher amino acid levels, which include alanine, GABA, aspartate, and glutamate (Figure S2).

## 4. Discussion

The findings of the present study indicated that variations in the secondary metabolite contents of different cultivars of Chinese cabbage have been reported in previous studies in which glucosinolates, carotenoids, and phenolics have been profiled and quantified. Chun et al., 2018 [16], for example, demonstrated that 13 glucosinolates were detected in 11 varieties of Chinese cabbage and the total glucosinolate levels in the different varieties were in the following order: “K0416” > “K0112” > “K0461” > “K0648” > “Chunkwang” > “CR Hakwang” > “K0015” > “BulamPlus” > “Bulam No. 3” > “K0588” > “K0651”. Similarly, the total glucosinolate contents varied significantly amongst 24 varieties of Chines cabbage (“Tsao Huang Pai”, “Hwiparam”, “Tschifu”, “Jangmi”, “Tae Tschong Bang”, “Zibu”, “Matchum”, “Taetschenpan”, “Noranja”, “TS 22”, “Gawulmat”, “Hwangseong”, “Hukjinju”, “Tung An Pai Tsai”, “Buram”, “Phoduran”, “Fan Hsin Huang”, “Digeson”, “CR-mat”, “Samjin”, “e-Norang”, “Sandun,” “BP 79” and “Kori”) and the “Kori”, “Sandun” and “e-Norang” cultivars seemed to be good for future breeding programs due to their high quantity of glucosinoaltes [17]. A total of 10 glucosinoaltes (sinigrin, progoitrin, gluconasturtiin, glucoalyssin, gluconapin, glucobrassicanapin, neoglucobrassicin, glucobrassicin, 4-methoxyglucobrassicin, and 4-hydroxyglucobrassicin) was identified and quantified in 62 varieties collected in South Korea and their total glucosinolate contents varied extensively [10]. Additionally, Baek et al., 2016 indicated that the contents of the phytochemicals, violaxantin, lutein, antheraxanthin, α-carotene, and β-carotene, varied amongst nine cultivars of Chinese cabbage (“Chuno”, “Cheongomabi”, “Cheonsangcheonha”, “Hwangkeummulgeul”, “Kangsimjang”, “Waldongcheonha” and “CRmat”) and the “Cheonsangcheonha” and “Waldongcheonha” cultivars contained the highest levels of carotenoids [18]. Also, the difference in carotenoid contents has been reported between Chinese cabbage (*B*. *rapa* cv. Orange queen) and Chinese cabbage (*B*. *rapa* cv. Yuki) [19]. Several studies have reported the accumulation of phenolic compounds in Chinese cabbage and that the phenolic contents of different Chinese cabbage cultivars show marked variation, which is consistent with our findings. The red Chinese cabbage (*B*. *rapa* cv. Shinhong Ssam) and green Chinese cabbage (*B*. *rapa* cv. Samjin) showed remarkable phenotypic difference derived from anthocyanins, belonging to phenolic compounds, and the red cultivar contained higher levels of caffeic acid, *p*-coumaric acid, ferulic acid, sinapic acid, quercetin, kaempferol, isorhamnetin, and anthocyanidins than the green cultivar [20]. Similarly, the different red Chinese cabbage cultivar (*B*. *rapa* cv. Kwonnongbbalgang No. 2) and Green Chinese cabbage cultivars (*B*. *rapa* cv. Bulam No. 3, *B*. *rapa* cv. Hwangsim, and *B*. *rapa* cv. CR-power Chunkwang) revealed the variations in the phenolic contents [21]. These variations in secondary metabolite contents are not surprising, as many factors are involved [22]. It might be ascribed to species specificity [23], variety specificity [24,25], environmental factors [26], climatic factors [27], or chemical analysis conditions [28] affecting the quality and quantity of the plant secondary metabolites.

Carbon and nitrogen are the most important nutrients for fundamental cellular functions. Carbohydrates, which contain carbon, play a main role in plant metabolism. Amino acids, one of the types of organic nitrogen compounds, are important building blocks required by plants to perform a variety of cellular activities and provide precursors for the synthesis of a range of secondary metabolites [29,30]. The metabolite map showed that the oval Chinese cabbage has higher pools of carbohydrates (represented mainly by xylose, glucose, mannose, fructose, mannitol, myo-inositol, glycerol, sucrose, raffinose, and maltose) than the rectangular Chinese cabbage, reflecting the higher production to meet carbon and energy demands for the biosynthesis of amino acids and secondary metabolites (phenylpropanoids and glucosinolates). Furthermore, the levels of TCA intermediates were also higher in the oval cultivar, suggesting that carbohydrate metabolism is more active in this cultivar than in the rectangular cultivar. The levels of glutamate and glutamine, as precursors for the synthesis of other amino acids, were also higher in the oval cultivar, suggesting that this cultivar is better able to assimilate nitrogen from the soil and provides more precursors for the production of other amino acids in comparison with the rectangular cultivar. Our results indicating that endogenous pools of carbon-abundant or nitrogen-abundant primary metabolites are likely to be correlated with the pools of carbon-abundant or nitrogen-abundant secondary metabolites are consistent with the findings of previous studies [31]. Zakhleniuk et al. [32] reported that the *Arabidopsis* mutant *pho3*, harboring a defective copy of the *Suc transporter* 2 gene, had large pools of glucose, fructose, sucrose, and starch, leading to retarded growth and enhanced anthocyanin accumulation. Additionally, an increased supply of exogenous sucrose has been observed to induce enhanced biosynthesis of phenylpropanoids in cell cultures of *Vitis vinifera* [33].

Shikimate metabolism leads to tryptophan, which is used as a precursor for indolic glucosinolate metabolism, and phenylalanine, which is used for phenylpropanoid metabolism [32]. In the present study, the rectangular cultivar of Chinese cabbage was observed to contain larger amounts of shikimate, which is consistent with the higher abundances of indolic glucosinolates (glucobrassicin, 4-hydroxy glucobrassicin, and neoglucobrassicin) in this cultivar. In contrast, the level of phenylalanine was higher in the oval cultivar, which is consistent with the observation that most phenylpropanoid compounds (cinnamate, ferulate, sinapate, *p*-hydroxybenzoic acid, rutin, chlorogenic acid, and catechin) were higher in this cultivar than the rectangular cultivar, based on *t*-test. Methionine, derived from aspartate, is a precursor for aliphatic glucosinolate metabolism. The oval cultivar contained a larger pool of aspartate, leading to higher contents of aliphatic glucosinolates (gluconapin, glucoiberin, and glucobrassicanapin). Previously, Sakuta et al. [34] reported that in *Vitis* cells the increased pool of endogenous phenylalanine, caused by cessation of cell division, may activate transcription of mRNAs of the enzymes phenylalanine ammonia-lyase (PAL) and chalcone synthase (CHS), and that the resulting increased activities of these enzymes resulted in the enhanced production of anthocyanin. Additionally, exogenous supply of phenylalanine induced an increased expression of *CHS* mRNA and accumulation of anthocyanin when the *Vitis* cell culture exhibited a low concentration of endogenous phenylalanine at the division stage. It has also been reported that the overexpression of *PAL*, involved in the conversion of phenylalanine to cinnamate, resulted in enhanced levels of phenylpropanoids in transformed tobacco [35]. Furthermore, other phenylpropanoid biosynthetic genes, cinnamate 4-hydroxylase (C4H) and chalcone isomerase (CHI), have been shown to be significantly correlated with accumulation of the corresponding phenylpropanoids [36,37].

## 5. Conclusions

This is the first study to profile primary and secondary metabolites in the oval and rectangular types of Chinese cabbage, and the results confirm that HPLC and GC-TOFMS-based metabolite profiling are appropriate techniques to determine metabolic differences and links in the different Chinese cabbage cultivars. A total of 42 primary metabolites (sugars, sugar alcohols, organic acids, amino acids, and an amine), eight glucosinolates, nine carotenoids, and nine phenolics were identified and quantified using HPLC and GC-TOFMS to comprehensively determine the relationship between primary and secondary metabolites in both cultivars. The oval type contained a higher amount of glucosinolates and phenolics, whereas levels of carotenoids were higher in the rectangular type. It is expected that the current information on the contents of glucosinolates, carotenoids, and phenolics in two cultivars used for human consumption will contribute to future databases. Furthermore, the information may prove useful for developing strategies to enhance the biosynthesis of different phytochemicals, particularly glucosinolates, phenolics, and carotenoids, in Chinese cabbage.

## Figures and Tables

**Table 1:** Glucosinolate contents in oval and rectangular Chinese cabbage.

Glucosinolate (μg g^−1^ Dry Weight)	Oval Chinese Cabbage	Rectangular Chinese Cabbage
Progoitrin	298.40 ± 3.89	300.07 ± 11.68
Glucoalyssin	123.5 ± 44.52 *^,1^	94.98 ± 13.55
Gluconapin	610.21 ± 7.47 ***	204.56 ± 0.00
4-hydroxyglucobrassicin	48.99 ± 9.29	109.73 ± 4.64 ***
Glucobrassicanapin	263.35 ± 3.87 ***	108.38 ± 0.00
Glucobrassicin	652.00 ± 22.42	935.06 ± 76.24 ***
4-methoxyglucobrassicin	767.00 ± 57.42	666.10 ± 43.06
Neoglucobrassicin	133.29 ± 4.78	158.92 ± 9.57 *
Total ^2^	2896.78 ± 113.67 *	2577.79 ± 158.74

^1^ Asterisks indicate significant differences (*t*-test, * *p* < 0.05, *** *p* < 0.005). ^2^ Total means the sum of 8 glucosinolates.

**Table 2:** Phenolic contents in oval and rectangular Chinese cabbage.

Phenolics (μg g^−1^ Dry Weight)	Oval Chinese Cabbage	Rectangular Chinese Cabbage
*p*-hydroxybenzoic acid	63.41 ± 3.58 **^,1^	47.94 ± 3.03
Catechin hydrate	11.34 ± 1.06	n.d ^2^
Chlorogenic acid	37.53 ± 0.75 *	34.86 ± 1.41
Caffeic acid	5.39 ± 0.39	8.00 ± 0.55 ***
Epicatechin	34.24 ± 7.42	38.17 ± 0.50
Ferulate	67.97 ± 1.42 ***	34.81 ± 1.16
Benzoic acid	n.d	29.97 ± 1.59
Rutin	5.11 ± 0.29	n.d
*t*-cinnamate	0.99 ± 0.22	0.59 ± 0.03
Total ^3^	226.53 ± 15.63 *	194.34 ± 4.94

^1^ Asterisks indicate significant differences (*t*-test, * *p* < 0.05, ** *p* < 0.01, *** *p* < 0.005). ^2^ n.d, not detected. ^3^ Total means the sum of nine phenolics.

**Table 3:** Carotenoid contents in oval and rectangular Chinese cabbage.

Carotenoid (μg g^−1^ Dry Weight)	Oval Chinese Cabbage	Rectangular Chinese Cabbage
Violaxanthin	82.17 ± 1.81	134.35 ± 3.19 ***^,1^
Antheraxanthin	24.42 ± 0.10	43.06 ± 0.13 ***
Lutein	350.06 ± 11.96	839.06 ± 9.20 ***
Zeaxanthin	12.34 ± 0.38	17.31 ± 0.24 ***
β-Cryptoxanthin	1.72 ± 0.00	5.27 ± 0.58 ***
13-*cis*-β-Carotene	8.04 ± 0.63	19.56 ± 1.77 ***
α-Carotene	2.81 ± 0.07	3.91 ± 0.53 *
β-Carotene	251.15 ± 5.65	561.94 ± 54.72 ***
9-*cis*-β-Carotene	9.04 ± 0.18	18.67 ± 2.15 ***
Total ^2^	741.73 ± 20.75	1,643.13 ± 72.48 ***

^1^ Asterisks indicate significant differences (*t*-test, * *p* < 0.05, *** *p* < 0.005). ^2^ Total means the sum of nine carotenoids.

**Table 4:** Chlorophyll contents in oval and rectangular Chinese cabbage.

Chlorophyll (μg g^-1^ Dry Weight)	Oval Chinese Cabbage	Rectangular Chinese Cabbage
Chlorophyll a	434.54 ± 23.91	460.47 ± 23.91
Chlorophyll b	325.42 ± 118.01	831.70 ± 110.20 ***^,1^
Total ^2^	836.48 ± 104.85	1332.22 ± 146.51 ***

^1^ Asterisks indicate significant differences (*t*-test, *** *p* < 0.005). ^2^ Total means the sum of chlorophyll a and chlorophyll b.

## Supplementary Materials

The following are available online at https://www.mdpi.com/2304-8158/8/11/587/s1, Figure S1: The chromatogram of metabolites obtained from and oval Chinese cabbage using GC-TOFMS and HPLC, Figure S2: Metabolite peak height differences of rectangular and oval Chinese cabbage using GC-TOFMS. X axis indicates relative intensity, Table S1: Differential metabolites between rectangular and oval Chinese cabbage derived from the PLS-DA model of GC-TOFMS analysis, Table S2: Morphological characteristics of oval and rectangular Chinese cabbage, Table S3: Metabolites identified in GC-TOFMS chromatograms of oval-type Chinese cabbage.
