# Supplementary material for: Comparative Phytochemical Analyses and Metabolic Profiling of Different Phenotypes of Chinese Cabbage (Brassica Rapa ssp. Pekinensis)
Source: Foods. 2019 Nov 19;8(11):587. doi: 10.3390/foods8110587 (PMC6915346; doi:10.3390/foods8110587)
Supplement: Supplementary file 1 [file foods-08-00587-s001.pdf]

Figure S1. The chromatogram of metabolites obtained from and oval shape Chinese cabbage using GC-TOFMS and HPLC.

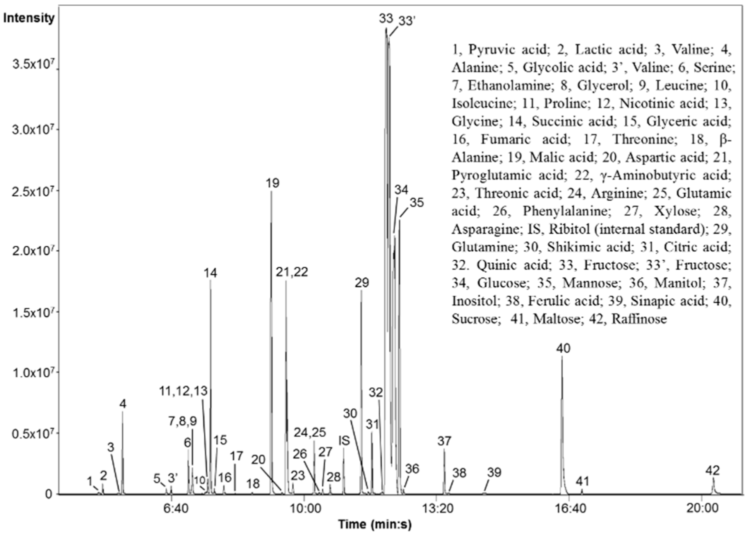

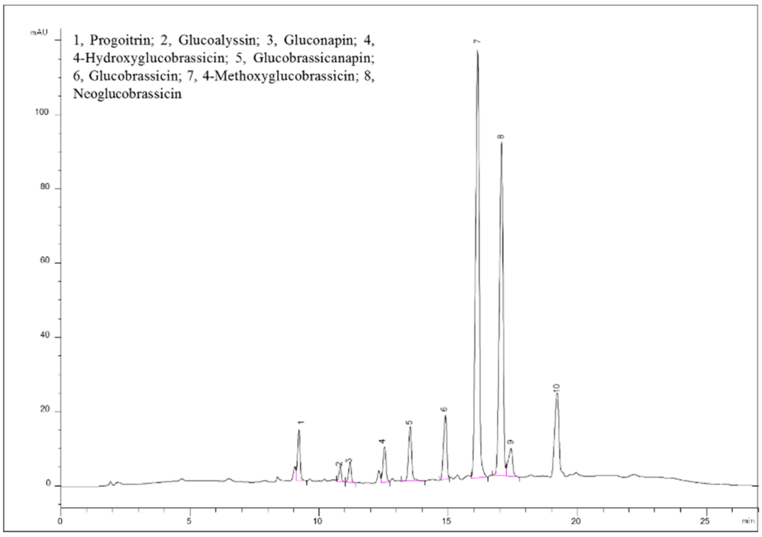

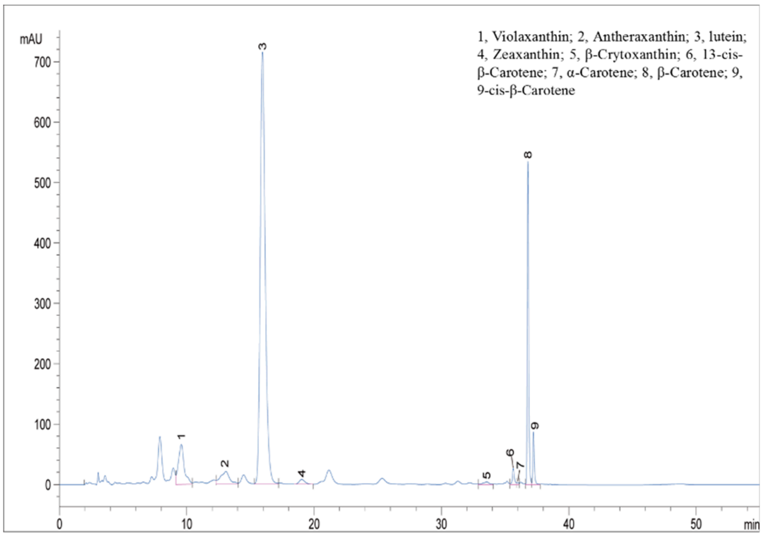

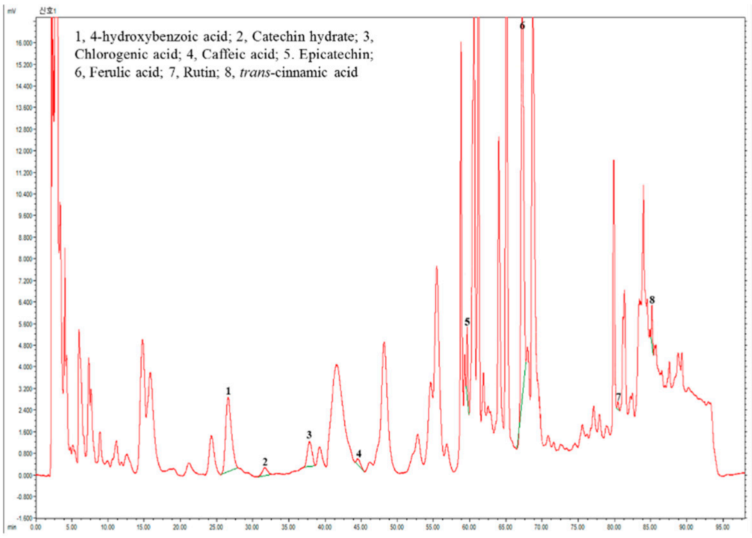

Figure S2. Metabolite peak height differences of rectangular and oval shape Chinese cabbage using GC-TOFMS. X axis indicates relative intensity. Asterisks indicate significant differences (*t*-test, * $p < 0.05$, ** $p < 0.01$, *** $p < 0.005$). .

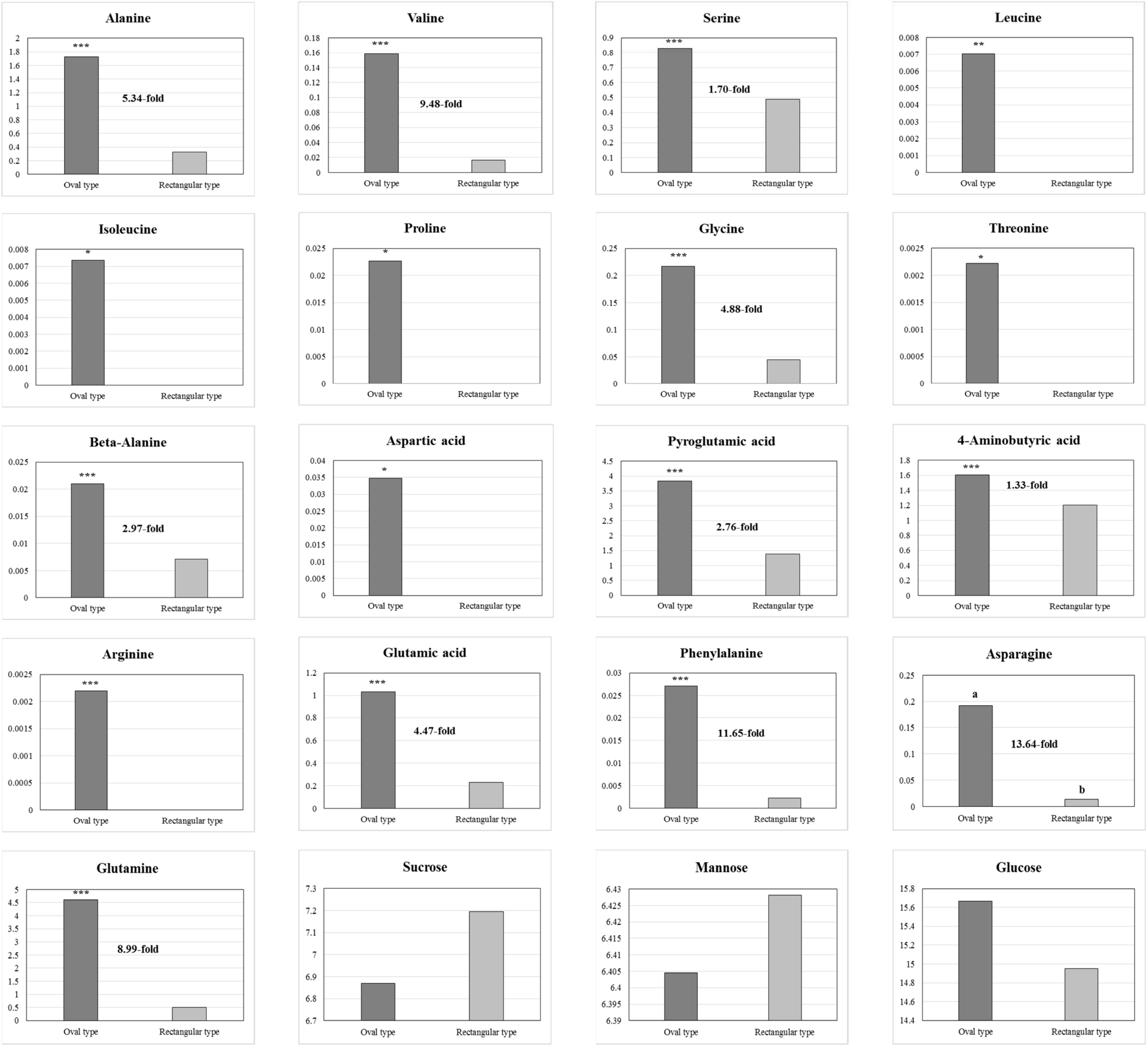

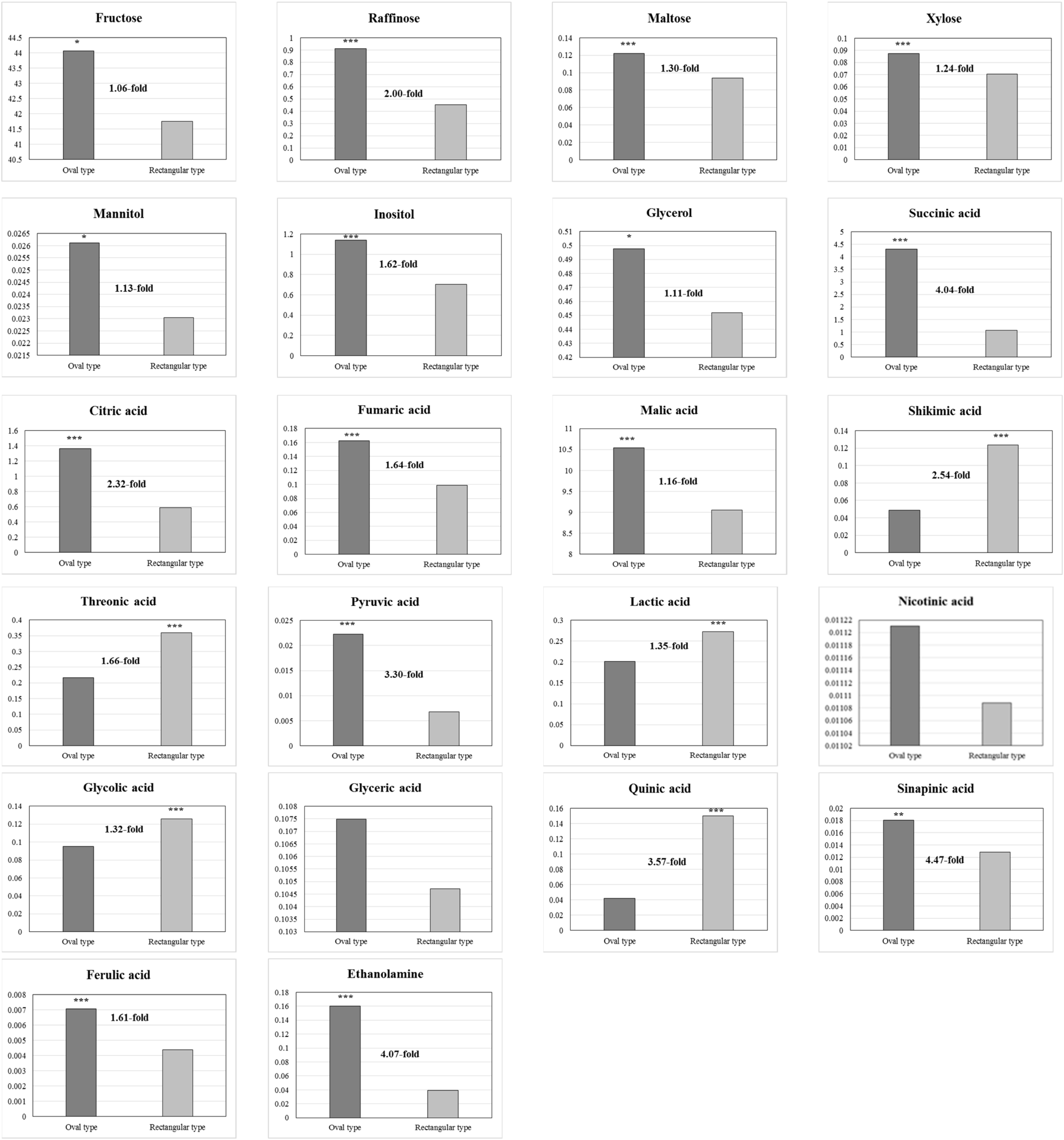

Fructose
*
1.06-fold
Raffinose
***
2.00-fold
Maltose
***
1.30-fold
Xylose
***
1.24-fold
Mannitol
*
1.13-fold
Inositol
***
1.62-fold
Glycerol
*
1.11-fold
Succinic acid
***
4.04-fold
Citric acid
***
2.32-fold
Fumaric acid
***
1.64-fold
Malic acid
***
1.16-fold
Shikimic acid
***
2.54-fold
Threonic acid
***
1.66-fold
Pyruvic acid
***
3.30-fold
Lactic acid
***
1.35-fold
Nicotinic acid
Glycolic acid
***
1.32-fold
Glyceric acid
Quinic acid
***
3.57-fold
Sinapinic acid
**
4.47-fold
Ferulic acid
***
1.61-fold
Ethanolamine
***
4.07-fold
Oval type
Rectangular type

Table S1. Differential metabolites between rectangular and oval shape Chinese cabbage derived from the PLS-DA model of GC-TOFMS analysis.

Metabolite	VIP †	*p* Value ‡	Fold Change §
Glycine	1.164	<0.0001	4.88±0.08
Succinic acid	1.164	<0.0001	4.04±0.08
Ethanolamine	1.164	<0.0001	4.09±0.36
Citric acid	1.163	<0.0001	2.32±0.10
Glutamine	1.163	<0.0001	9.33±2.02
β-Alanine	1.163	<0.0001	2.98±0.18
Serine	1.163	<0.0001	1.70±0.02
Asparagine	1.162	<0.0001	13.90±2.47
Glutamic acid	1.162	<0.0001	4.57±0.86
Raffinose	1.162	<0.0001	2.01±0.05
Inositol	1.161	<0.0001	1.62±0.06
Quinic acid	1.161	0.0015	0.28±0.01
Fumaric acid	1.161	<0.0001	1.64±0.03
Threonic acid	1.160	<0.0001	0.60±0.01
Phenylalanine	1.159	<0.0001	12.10±2.80
Shikimic acid	1.158	<0.0001	0.39±0.02
Maltose	1.156	<0.0001	1.30±0.04
Valine	1.155	<0.0001	9.64±1.59
Xylose	1.154	0.0001	1.24±0.01
Alanine	1.154	0.0001	7.12±5.21
4-Aminobutyric acid	1.153	0.0001	1.33±0.03
Glycolic acid	1.144	0.0005	0.76±0.02
Pyruvic acid	1.143	0.0005	3.31±0.34
Malic acid	1.140	0.0007	1.16±0.03
Pyroglutamic acid	1.138	0.0112	2.77±0.37
Ferulic acid	1.122	0.0021	1.64±0.23
Lactic acid	1.098	0.0048	0.74±0.07
Glycerol	1.063	0.0427	1.10±0.04
Sinapinic acid	1.060	0.0122	1.44±0.25

† Variable importance in the projection (VIP) was obtained from the PLS-DA model with a threshold of 1.0. ‡ *P* value obtained from Student's *t*-test. § Fold change between rectangle and oval type. The values are expressed relative to rectangle type and presented as the mean ± SD of determinations from three independent samples.

Table S2. Morphological characteristics of oval and rectangular shape Chinese cabbage.

Phenotype	Height (cm)	Width (cm)	Number of Leaves	Head Weight (kg)	Color
Oval shape Chinese cabbage	25.25 ± 3.86	18.75± 1.50***	64.00 ± 6.58***	2.21 ± 0.23	Green
Rectangular shape Chinese cabbage	48.80 ± 1.64***[1]	12.20 ± 1.10	39.60 ± 2.61	2.12 ± 0.23	Deep green

[1] Asterisks indicate significant differences (*t*-test, * $p < 0.05$, ** $p < 0.01$, *** $p < 0.005$).

Table S3. Metabolites identified in GC-TOFMS chromatograms of oval-type Chinese cabbage.

Compound	RT†	RRT‡	Selected Ion for Quantification§	Mass Fragment#
Pyruvic acid	4.835	0.440	174	115, 174, 189
Lactic acid	4.933	0.449	147	117, 147, 191
Valine	5.330	0.485	146	130, 146, 156
Alanine	5.433	0494	116	116, 147, 190
Glycolic acid	6.383	0.580	147	147, 177, 205
Serine	7.098	0.645	116	116, 132, 147
Ethanolamine	7.180	0.653	174	100, 147, 174
Glycerol	7.193	0.654	147	103, 117, 147
Leucine	7.215	0.656	158	102, 147, 158
Isoleucine	7.432	0.676	158	147, 158, 218
Proline	7.518	0.684	142	142, 158, 216
Nicotinic acid	7.550	0.687	180	106, 136, 180
Glycine	7.577	0.689	174	147, 174, 248
Succinic acid	7.645	0.695	147	129, 147, 247

Glyceric acid	7.747	0.704	147	133, 147, 189
Fumaric acid	7.983	0.726	245	143, 147, 245
Threonine	8.268	0.752	219	101, 117, 219
β-Alanine	8.695	0.791	174	147, 174, 248
Malic acid	9.182	0.835	147	147, 233, 245
Aspartic acid	9.448	0.859	100	100, 147, 232
Pyroglutamic acid	9.550	0.868	156	147, 156, 230
4-Aminobutyric acid	9.578	0.871	174	147, 174, 304
Threonic acid	9.722	0.884	147	147, 205, 220
Arginine	10.223	0.930	142	142, 147, 162
Glutamic acid	10.257	0.933	246	128, 156, 246
Phenylalanine	10.390	0.945	218	100, 192, 218
Xylose	10.470	0.952	103	103, 147, 217
Asparagine	10.657	0.969	116	116, 132, 231
IS	10.997	1.000	217	103, 147, 217
Glutamine	11.443	1.041	156	147, 156, 245
Shikimic acid	11.618	1.056	204	147, 204, 255
Citric acid	11.710	1.065	273	147, 273, 347
Quinic acid	11.972	1.089	345	147, 255, 345
Fructose	12.057	1.096	103	103, 147, 217
Glucose	12.285	1.117	147	147, 160, 205
Mannose	12.402	1.128	147	147, 205, 319
Manitol	12.500	1.137	319	147, 217, 319
Inositol	13.532	1.231	305	147, 217, 305
Ferulic acid	13.633	1.240	338	308, 323, 338
Sinapic acid	14.527	1.321	338	338, 353, 368
Sucrose	16.497	1.500	217	147, 217, 361
Maltose	16.992	1.545	147	147, 204, 361
Raffinose	20.302	1.846	217	204, 217, 361

[†] Retention time (min). [‡] Relative retention time (retention time of the analyte/retention time of the IS). [§] Specific mass ion used for quantification. [#] First three ions with the highest intensities.
